# Progressive Pulmonary Lesions and Cervical Lymphadenopathy Revealing Large B-cell Lymphoma: A Rare Case Report

**DOI:** 10.7759/cureus.106723

**Published:** 2026-04-09

**Authors:** Muhammad A Tariq, Blessing T Ojinna, Efrah Ashraf, Arshia Ahmed, Salman J Khan, Zia H Shah, Gurdeep Singh

**Affiliations:** 1 Internal Medicine, Guthrie Lourdes Hospital, Binghamton, USA; 2 Medicine and Surgery, Quaid-e-Azam Medical University, Bahawalpur, PAK; 3 Global Health, Emory University, Rollins School of Public Health, Atlanta, USA; 4 Public Health, University of Massachusetts, Amherst, USA; 5 Endocrinology, Diabetes, and Metabolism, Guthrie Lourdes Hospital, Binghamton, USA

**Keywords:** cervical lymphadenopathy, diffuse large b cell lymphoma (dlbcl), dlbcl, hematology, oncology, pulmonary lesions

## Abstract

Primary pulmonary and thyroid diffuse large B-cell lymphoma (DLBCL) is an exceptionally rare extranodal manifestation of non-Hodgkin lymphoma and often presents with nonspecific clinical and radiographic features, leading to significant diagnostic challenges. We report a case of a 70-year-old woman with extensive past medical history who was incidentally found to have pulmonary abnormalities during routine abdominal magnetic resonance imaging (MRI) surveillance for hepatocellular carcinoma. Subsequent non-contrast computed tomography of the chest revealed multiple spiculated pulmonary masses involving the left upper and lower lobes and a nodular lesion at the right lung base. Positron emission tomography/computed tomography demonstrated marked fluorodeoxyglucose avidity within these pulmonary lesions, as well as focal uptake in the left thyroid lobe. Despite the absence of constitutional “B” symptoms, malignancy was strongly suspected. The initial pathology from the left lower lobe lung biopsy showed only benign features consistent with organizing pneumonia. Given the possibility of false negative results, further evaluation with ultrasound-guided fine-needle aspiration of a left cervical mass adjacent to the thyroid gland revealed an atypical lymphoid population suspicious for large B-cell lymphoma. A repeat lung biopsy ultimately confirmed DLBCL, with immunohistochemistry positive for CD20 and negative for CD3 and cytokeratin. This case underscores the diagnostic complexity of primary extranodal DLBCL involving the lung and thyroid, highlights the limitations of initial tissue sampling, and emphasizes the importance of persistent diagnostic evaluation and multidisciplinary collaboration when clinical suspicion remains high.

## Introduction

Lymphoma is a malignancy of lymphocytes within the lymphoid system. These cancers can originate from B lymphocytes, T lymphocytes, or natural killer (NK) cells at various stages of maturation. B cells exhibit significant functional diversity and can differentiate along multiple pathways [1].

Diffuse large B-cell lymphoma (DLBCL) is an aggressive B-cell lymphoma that is histologically defined by the diffuse proliferation of large neoplastic B lymphoid cells. These cells possess nuclei that are equal to or larger in size than those of normal histiocytes [2]. DLBCL is the most prevalent form of lymphoma, accounting for approximately 25% to 30% of all non-Hodgkin lymphomas. It commonly manifests as a rapidly enlarging mass or lymphadenopathy at nodal or extranodal sites. Although DLBCL is aggressive, it often responds favorably to six cycles of rituximab combined with cyclophosphamide, doxorubicin, vincristine, and prednisone (R-CHOP) [3]. Pulmonary involvement in DLBCL can be classified as either primary pulmonary DLBCL (PP-DLBCL) or secondary. PP-DLBCL, which arises directly from lung tissue, is exceptionally rare, accounting for less than 10% of all primary pulmonary lymphomas (PPLs) and only 0.4% of all lymphomas. In contrast, secondary pulmonary DLBCL (SP-DLBCL), which originates at an extrapulmonary site and subsequently involves the lung, is far more common and accounts for the majority of pulmonary DLBCL cases. PP-DLBCL is generally defined as lymphoma confined to the lung parenchyma, with or without hilar lymph node involvement, in the absence of extrapulmonary disease at the time of diagnosis or within three months thereafter. PP-DLBCL tends to occur most frequently in older adults (median age around 60 years) or in immunosuppressed individuals [4].

Similarly, thyroid involvement by lymphoma is uncommon, representing a small fraction of extranodal lymphomas, and is often associated with underlying autoimmune thyroiditis. Primary thyroid lymphoma (PTL) is an uncommon malignancy, comprising 1%-5% of all thyroid cancers and approximately 2%-5% of extranodal lymphomas, with a higher incidence in women compared to men. PTL is typically aggressive and often presents as a rapidly enlarging neck mass. Nearly 98% of PTLs are non-Hodgkin B-cell lymphomas, with DLBCL being the most prevalent subtype (60%-70%) and presenting with a shorter duration of symptoms before diagnosis. Fine-needle aspiration cytology (FNAC), usually performed after ultrasound evaluation, is the first-line diagnostic tool for thyroid nodules [5-6].

The simultaneous presentation of pulmonary and thyroid lesions as the initial manifestation of DLBCL is exceptionally uncommon and presents a significant diagnostic challenge. We present a case of DLBCL initially manifested by pulmonary lesions in conjunction with a thyroid mass, underscoring the importance of including lymphoma in the differential diagnosis of atypical pulmonary and thyroid presentations. This case emphasizes the pivotal roles of histopathological examination and immunohistochemistry in confirming the diagnosis and underscores the need for greater clinical awareness of rare extranodal manifestations of DLBCL.

## Case presentation

**Figure 1:**
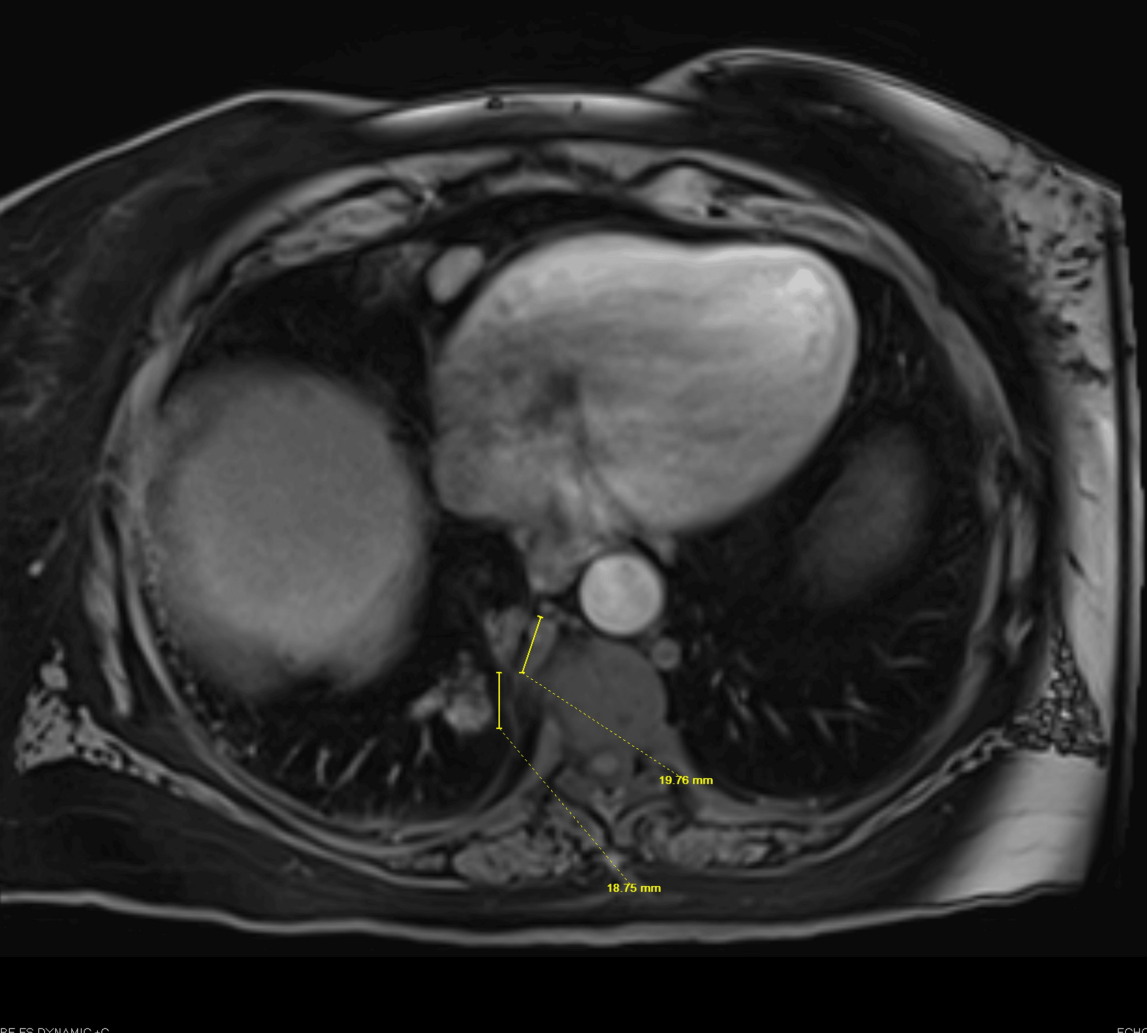
MRI showing incidental nodular lesions yellow arrows in the right lower lobe of the lung MRI: magnetic resonance imaging

**Figure 2:**
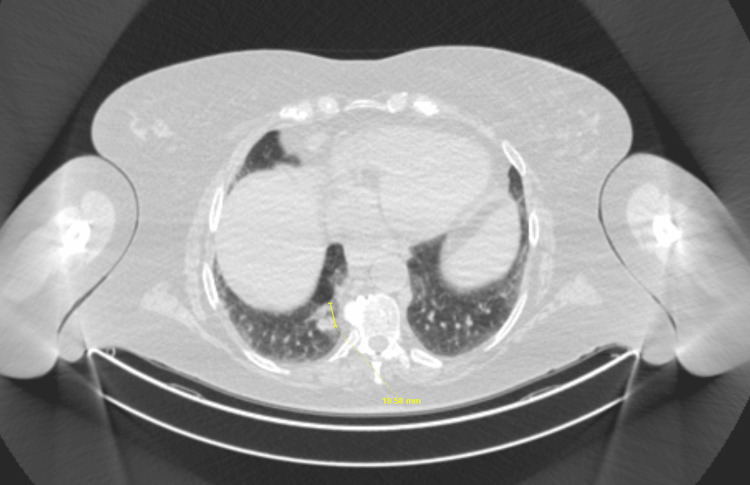
Corresponding CT scan with a yellow arrow showing incidental nodular lesions in the right lower lobe of the lung CT: computed tomography

A 70-year-old woman with extensive past medical history of hypertension, hyperlipidemia, hypothyroidism, type 2 diabetes mellitus, heart failure with preserved ejection fraction, coronary artery disease, nonalcoholic steatohepatitis (NASH)-related cirrhosis complicated by portal hypertension, grade III esophageal varices status post endoscopic band ligation, portal hypertensive gastropathy, idiopathic splenomegaly with associated chronic pancytopenia, stage 3b chronic kidney disease with anti-neutrophil cytoplasm antibodies (ANCA) positivity, Parkinson’s disease, depression, class II obesity (BMI 37.7 kg/m²) and obstructive sleep apnea, was referred to the pulmonology clinic by gastroenterology for evaluation of abnormal chest imaging. Surveillance MRI for NASH cirrhosis revealed multiple sub-centimeter arterially enhancing foci in the right hepatic lobe, concerning for regenerative or dysplastic nodules versus early hepatocellular carcinoma. Additionally, nodular lesions in the right lower lobe of the lung were identified (Figure 1), prompting a CT chest and subsequent referral to pulmonology (Figure 2). Her oncologic history included previously treated perinasal basal cell carcinoma, last excised approximately one year prior.

At the time of evaluation at the pulmonology clinic, the patient had a four-month history of progressive fatigue and a nonproductive cough. She denied constitutional B symptoms, and physical examination was unremarkable. Laboratory evaluation demonstrated pancytopenia, including leukopenia, absolute neutropenia, and lymphopenia. Normocytic anemia was present. Thrombocytopenia was noted. Inflammatory markers were essentially normal, and QuantiFERON-TB testing was negative. Thyroid function testing revealed a low thyroid-stimulating hormone (TSH) of 0.26 µIU/mL with a free T4 of 1.5 ng/dL while on levothyroxine 275 µg daily. Liver and renal function were within normal limits (Table 1).

**Table 1:** Laboratory findings ALT: alanine transaminase; AST: aspartate transaminase; CRP: C-reactive protein; ESR: erythrocyte sedimentation rate; TSH: thyroid-stimulating hormone; WBC: white blood cell

Laboratory Test	Reference Range	Results
WBC (K/µL)	2.16	3.98-10.04
Absolute neutrophils (ANC) (K/µL)	1.22	1.56-6.13
Lymphocytes (%)	25.9	19.3-51.7
Hemoglobin (g/L)	9.9	11.2-15.7
Hematocrit (%)	31.9	34.1-44.9
Platelet count (K/µL)	67	182-369
ESR (mm/hour)	32	0-30
CRP (mg/dL)	0.44	0.00-0.50
TSH (µIU/mL)	0.26	0.27-4.20
Free T4 (ng/dL)	1.5	0.7-1.7
ALT (U/L)	13	5-33
AST (U/L)	20	0-32
Creatinine (mg/dL)	1.14	0.51-0.95

**Figure 3:**
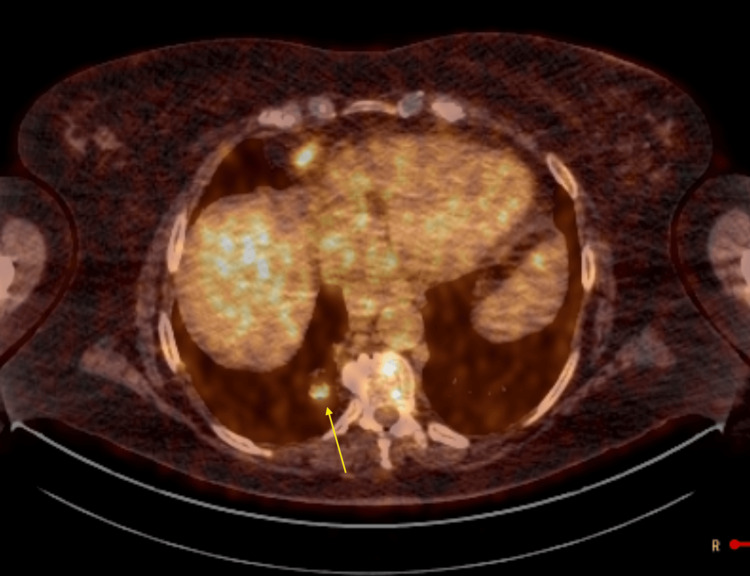
Corresponding PET scan showing an incidental nodular lesion in the RLL lung, as indicated by a yellow arrow PET: positron emission tomography; RLL: right lower lobe

**Figure 4:**
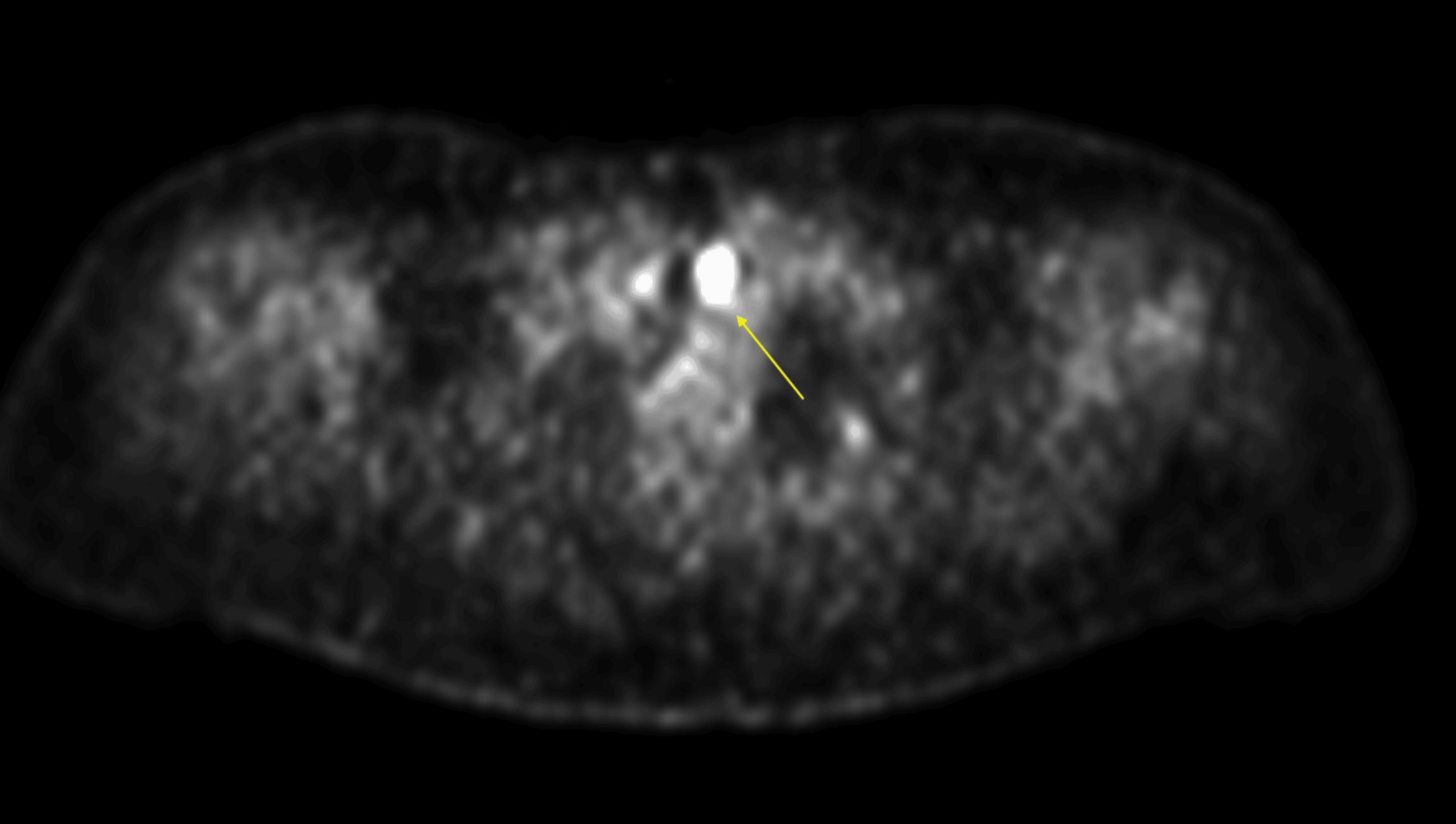
PET scan demonstrating FDG uptake in the left thyroid lobe, as indicated by a yellow arrow FDG: fluorodeoxyglucose; PET: positron emission tomography

**Figure 5:**
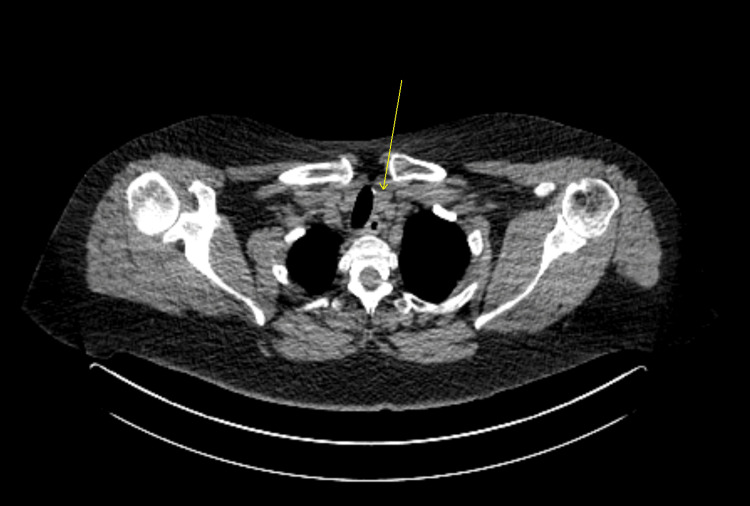
Increased metabolic activity in the left thyroid lobe on PET/CT scan, as indicated by a yellow arrow CT: computed tomography; PET: positron emission tomography

Given the presence of pancytopenia and concern for an underlying lymphoproliferative disorder, a whole-body PET scan was performed to assess disease extent and metabolic activity. PET imaging demonstrated multiple hypermetabolic pulmonary masses, including involvement of the left upper and left lower lobes and a mass-like lesion in the right lower lobe (Figure 3), with SUVmax up to 28.9. Incidental FDG uptake was also noted in the left thyroid lobe (Figures 4-5) and at the gastroesophageal junction, raising concern for extrapulmonary disease involvement.

**Figure 6:**
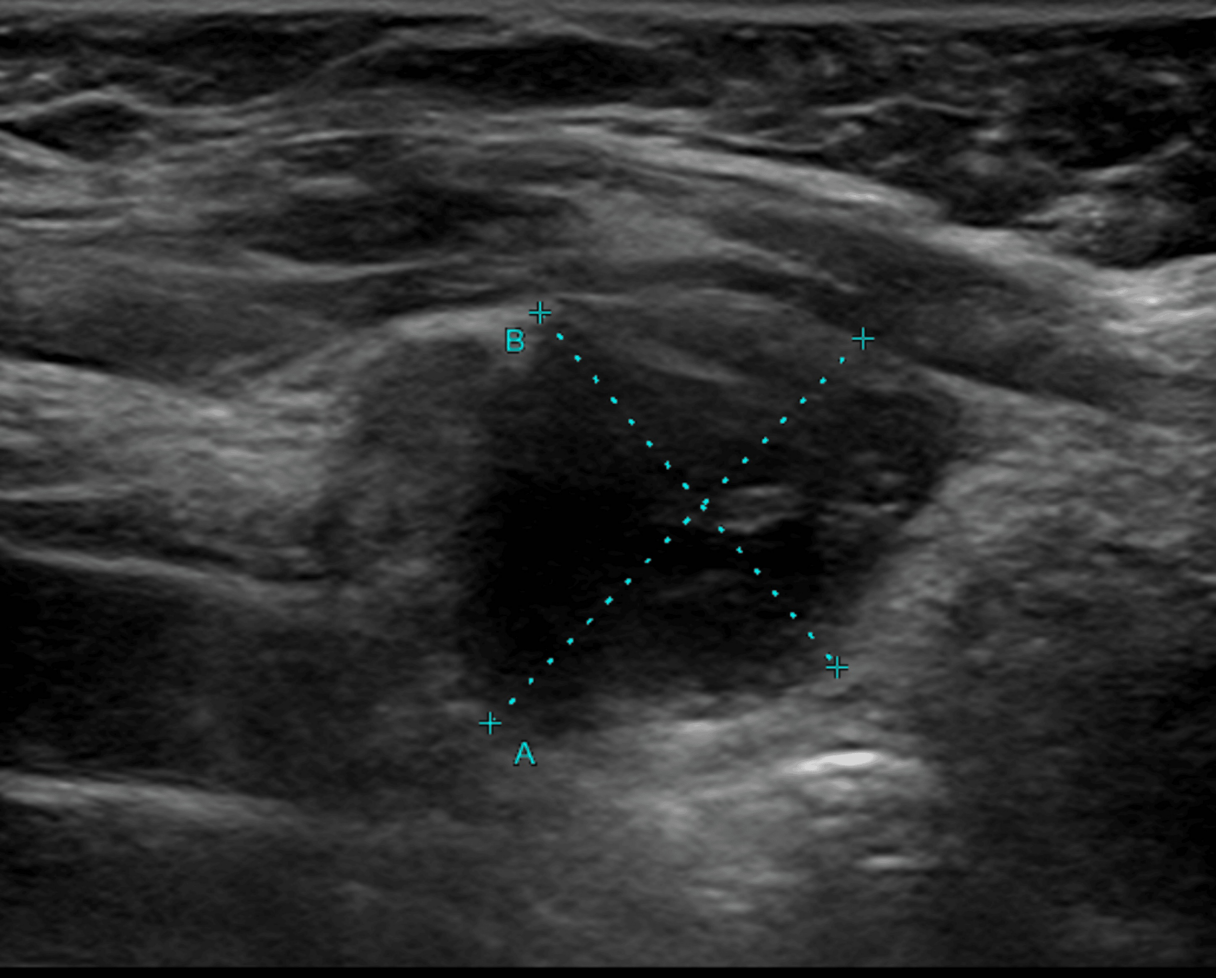
US thyroid showing interval appearance of the left thyroid nodule area marked with blue dotted lines corresponding to a hypermetabolic lesion on comparison PET CT CT: computed tomography; PET: positron emission tomography; US: ultrasound

**Figure 7:**
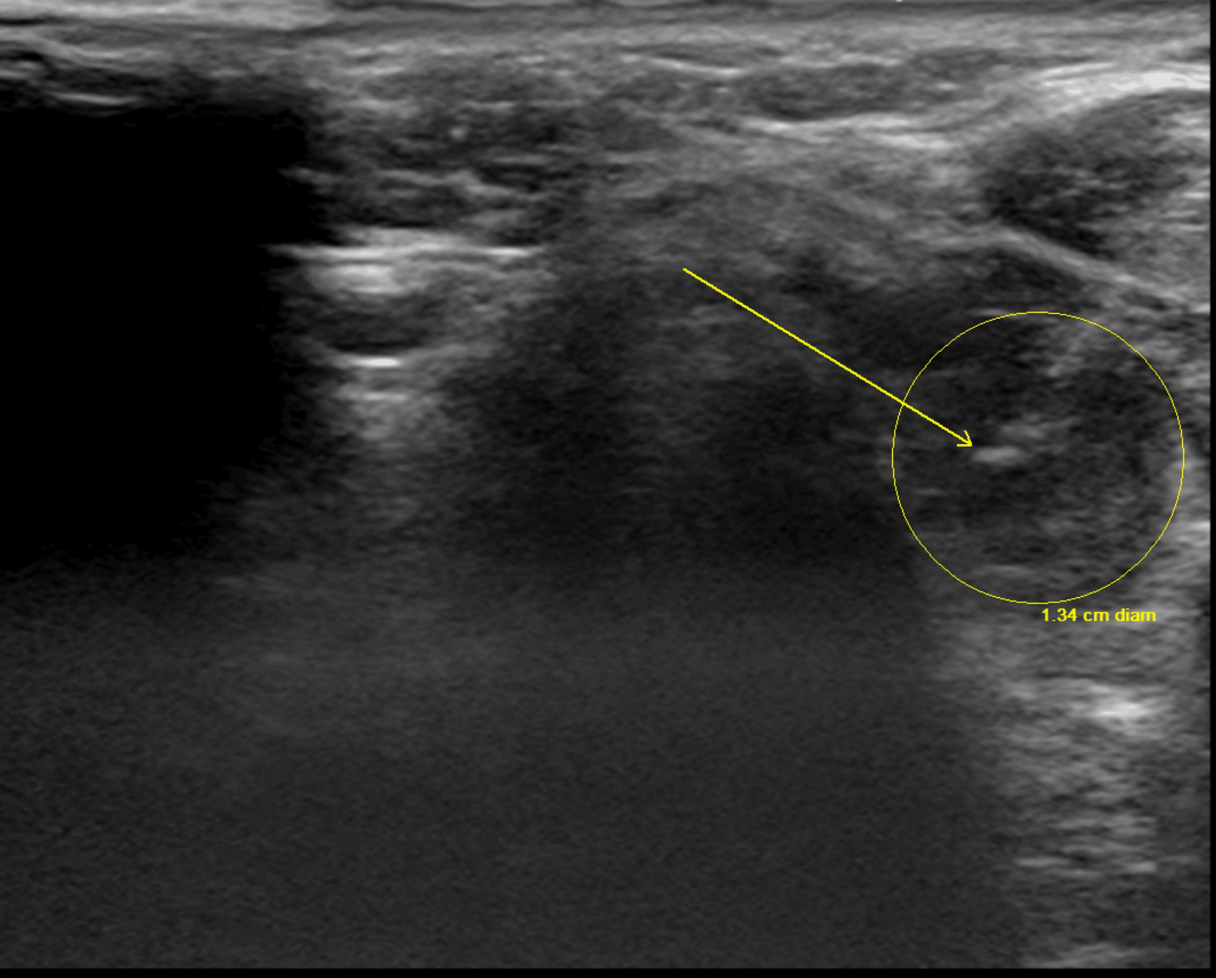
Demonstration of US-guided thyroid FNA biopsy, area marked in yellow FNA: fine needle aspiration; US: ultrasound

An initial CT-guided left lower lobe lung biopsy showed findings suggestive of organizing pneumonia, including interstitial fibrosis, intra-alveolar fibrin with macrophages, and focal interalveolar pneumocyte proliferation without cytologic atypia. Neck ultrasound demonstrated a well-circumscribed, heterogeneously hypoechoic mid-pole lesion measuring 2.4 × 2.1 × 1.5 cm without internal vascularity, corresponding to a hypermetabolic focus on PET/CT (Figure 6). Although initially interpreted as a thyroid nodule, repeat thyroid ultrasound during ultrasound-guided fine-needle aspiration revealed the lesion to be extrathyroidal, with cytology suspicious for DLBCL, consistent with an adjacent lymph node rather than a primary thyroid malignancy (Figure 7). 

**Figure 8:**
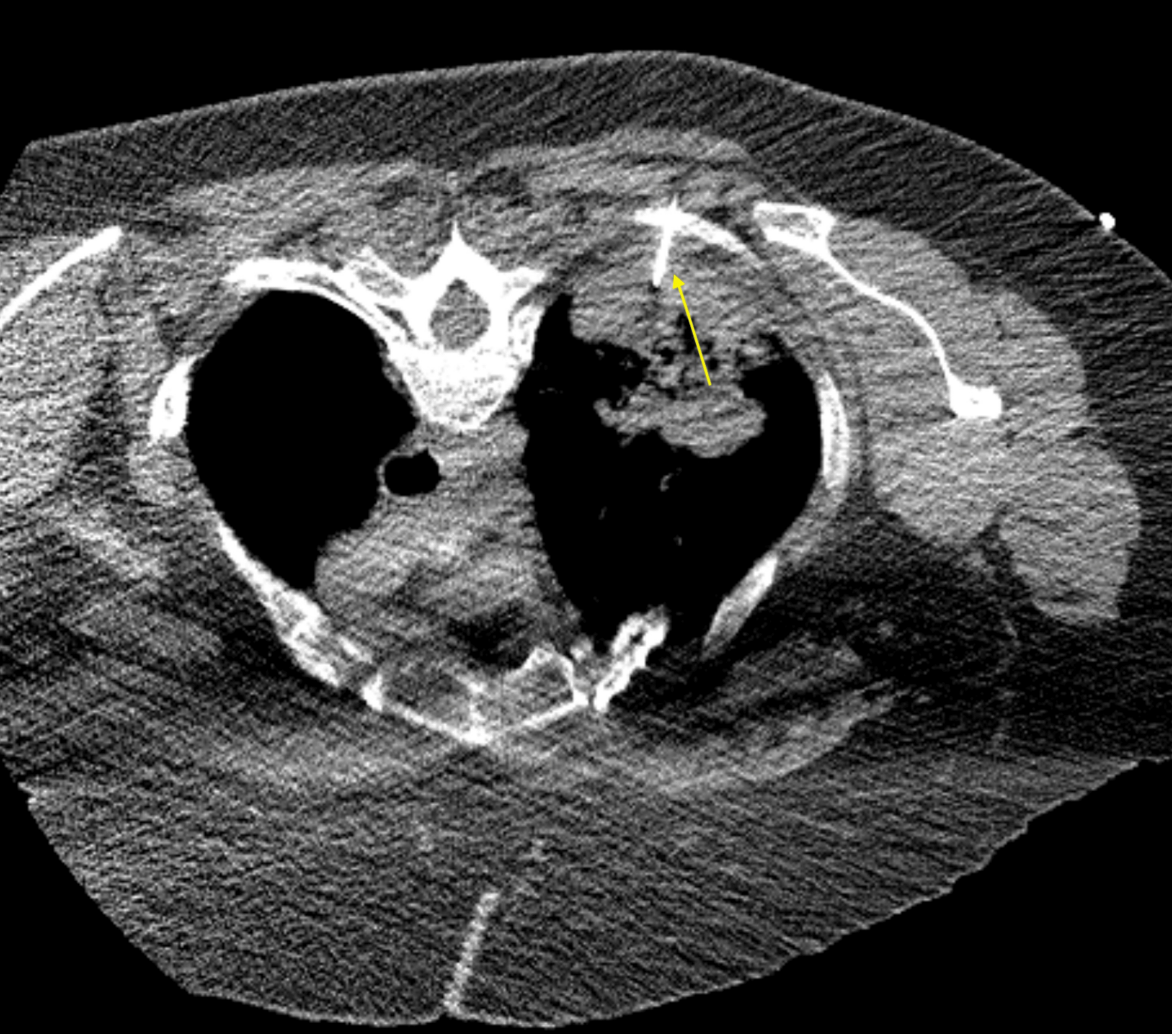
Demonstration of repeated CT-guided biopsy of a left upper lobe lung lesion, as indicated by a yellow arrow CT: computed tomography

Given the concern for underlying malignancy, a repeat CT-guided biopsy of a left upper lobe lung lesion was performed and demonstrated extensive necrosis (Figure 8). Immunohistochemical staining showed tumor cells positive for CD20 and negative for CD3 and cytokeratin, confirming the diagnosis of primary pulmonary DLBCL. 

After establishing the diagnosis, the patient was initiated on combination chemotherapy with R-CHOP, the standard first-line therapy for DLBCL. 

## Discussion

Large B-cell lymphoma (LBCL), most commonly DLBCL, is the most frequent subtype of non-Hodgkin lymphoma in adults, characterized by aggressive clinical behavior, marked biologic heterogeneity, and typically presents with rapidly enlarging lymphadenopathy, though extranodal involvement is common and may affect virtually any organ system [7]. The lung and thyroid are recognized but relatively uncommon sites of primary or secondary involvement, which can complicate timely diagnosis, particularly when clinical and radiographic findings overlap with more prevalent malignancies [8-9].

Pulmonary involvement in LBCL is rare. PPL accounts for less than 1% of all primary lung malignancies and approximately 3-4% of extranodal lymphomas, with the majority being indolent mucosa-associated lymphoid tissue (MALT) lymphomas [10-11]. In contrast, primary pulmonary LBCL represents a much smaller subset and tends to present aggressively with rapidly enlarging masses, constitutional symptoms, or respiratory compromise [10]. Radiographically, pulmonary LBCL often appears as solitary or multiple lung masses, nodules, or areas of consolidation, frequently accompanied by mediastinal lymphadenopathy [11]. These findings are nonspecific and commonly mimic primary lung carcinoma, metastatic disease, or infectious processes, especially in patients with underlying comorbidities or immunocompromised states [10-11]. As illustrated in this case, initial CT-guided lung biopsy may be nondiagnostic due to sampling error, necrosis, or heterogeneous tumor architecture, necessitating repeat or alternative biopsy approaches to establish a definitive diagnosis [10].

False-negative and nondiagnostic CT-guided lung biopsies represent a well-recognized diagnostic challenge in pulmonary LBCL, with nondiagnostic rates ranging from 6.8% to 16.7%, and up to 36-59% of these ultimately proving malignant on subsequent workup [12]. Multiple factors contribute to initial biopsy failure, including tumor-related factors such as extensive intratumoral necrosis (particularly common in large, rapidly growing aggressive lymphomas) and heterogeneous tumor architecture that may yield admixed inflammatory or fibrotic components rather than diagnostic lymphomatous tissue [12]. Technical factors include small lesion size (≤15 mm), needle trajectory traversing emphysematous parenchyma, prolonged procedure time, intraprocedural complications, and inadequate specimen volume (fewer than two cores) [12]. The clinical implications are substantial, as diagnostic delay in aggressive lymphoma can permit disease progression and narrow the therapeutic window. When the initial biopsy is nondiagnostic, strategies to optimize diagnostic yield include obtaining PET/CT prior to biopsy to guide needle placement toward metabolically active tumor regions, acquiring at least two tissue cores using core needle biopsy (≥18 gauge), and considering surgical biopsy when percutaneous approaches remain nondiagnostic [12].

Diagnosing PPL presents significant challenges due to its rarity and nonspecific clinical, radiographic, and histopathologic features. Patients often exhibit minimal or absent symptoms, and constitutional “B” symptoms are frequently lacking, which may delay clinical suspicion. Radiographically, PPL demonstrates a wide spectrum of imaging findings, including solitary or multiple pulmonary nodules, masses, or areas of consolidation, often mimicking more common conditions such as infection, organizing pneumonia, inflammatory lung disease, or primary lung carcinoma. Although positron emission tomography may demonstrate high fluorodeoxyglucose uptake, this finding is not specific and can overlap with infectious or inflammatory processes. Histopathologic diagnosis is further complicated by the frequent coexistence of inflammatory changes, fibrosis, or extensive necrosis, which may obscure malignant lymphoid cells and result in nondiagnostic or misleading initial biopsy results. Small tissue samples obtained through transbronchial or percutaneous biopsies are often insufficient for definitive diagnosis, limiting the ability to perform comprehensive immunohistochemical analysis and molecular studies required to distinguish lymphoma from reactive lymphoid infiltrates or poorly differentiated carcinoma [10,13]. Additionally, establishing the diagnosis of PPL requires exclusion of extrapulmonary disease, necessitating extensive staging and further complicating the diagnostic process. Collectively, these factors contribute to diagnostic delay and underscore the importance of maintaining a high index of suspicion and pursuing repeat or more extensive tissue sampling when initial evaluations are inconclusive [10,13]. 

Fujioka et al. described a case of PP-DLBCL that radiographically mimicked metastatic disease, presenting with multiple pulmonary nodules. In contrast, our patient initially demonstrated bilateral pulmonary masses with gradual progression of a single lesion among them over time, highlighting a less typical radiographic pattern and further contributing to diagnostic uncertainty [14]. 

Similarly, Li et al. reported a case of PP-DLBCL that was initially misdiagnosed as organizing pneumonia due to its radiologic appearance. While our patient’s pulmonary lesions appeared as consolidated masses on CT imaging, she lacked clinical features suggestive of pneumonia, such as respiratory symptoms or infectious markers, underscoring the variable and nonspecific imaging manifestations of PP-DLBCL [15]. 

The diagnosis of PTL requires a high index of suspicion and a structured approach integrating clinical presentation, imaging, and tissue-based evaluation. Thyroid involvement by LBCL is rare, accounting for less than 5% of thyroid malignancies and approximately 2% of extranodal lymphomas, and most commonly arises in the setting of chronic autoimmune thyroiditis, although it may occur in its absence [16-17]. Patients typically present with a rapidly enlarging thyroid or neck mass, compressive symptoms, or incidental imaging abnormalities [16-17]. Initial evaluation relies on high-resolution thyroid ultrasound, which often demonstrates a hypoechoic, heterogeneous mass or diffuse glandular enlargement with increased vascularity [16]. Ultrasound-guided FNAC is the preferred first-line diagnostic modality; however, its diagnostic yield is limited by overlap with chronic lymphocytic thyroiditis or reactive lymphoid hyperplasia [16]. Consequently, suspicious or nondiagnostic cytology frequently necessitates core needle biopsy or excisional sampling to obtain sufficient tissue for histopathologic assessment and immunohistochemical analysis, which is essential for confirming lymphoid origin and subtype, typically demonstrating B-cell markers such as CD20 [16-17]. Comprehensive staging with positron emission tomography-computed tomography is required to exclude systemic disease and establish primary thyroid involvement [17]. In our patient, fine-needle aspiration of the thyroid gland did not reveal cytologic atypia, and subsequent imaging and biopsy clarified that the hypermetabolic lesion represented an anterior cervical lymph node rather than primary thyroid pathology, ultimately confirming LBCL. This case underscores the diagnostic complexity of thyroid-region lymphomas and the importance of thorough tissue evaluation to guide timely and appropriate management. 

The standard treatment for LBCL, including DLBCL, is systemic immunochemotherapy, most commonly rituximab combined with anthracycline-based regimens such as R-CHOP. This approach achieves high response and cure rates in the general population. However, management becomes significantly more complex in patients with advanced liver disease and multiple comorbidities, as in this case of extranodal LBCL with concurrent pulmonary and cervical involvement [18]. 

The treatment strategy regarding extranodal LBCL with concurrent pulmonary and cervical involvement does not require fundamentally different systemic therapy; standard R-CHOP or pola-R-CHP remains appropriate. However, the presence of multiple extranodal sites contributes to higher NCCN-IPI risk scores, which may influence treatment intensity decisions [18]. 

Hepatic dysfunction from cirrhosis may limit the use or dosing of hepatically metabolized agents, particularly anthracyclines and vinca alkaloids, due to increased risk of toxicity. Baseline cytopenias related to portal hypertension and hypersplenism further complicate treatment, increasing susceptibility to infection, bleeding, and treatment-related myelosuppression. Cardiac comorbidities, such as congestive heart failure, may also preclude or necessitate modification of anthracycline-containing regimens. In such cases, dose-adjusted therapy, alternative non-anthracycline regimens, or substitution with less cardiotoxic agents may be considered [19].

Supportive care is a critical component of treatment, including careful monitoring of liver function, proactive management of cytopenias, infection prophylaxis, and close collaboration among oncology, hepatology, and cardiology teams. Localized radiotherapy may have a role for symptomatic extranodal disease or residual masses, but is generally adjunctive rather than curative in disseminated LBCL. 

Despite these challenges, treatment decisions should balance disease aggressiveness with the patient’s functional status and comorbidity burden, as LBCL remains a potentially curable malignancy. Individualized, multidisciplinary treatment planning is essential to optimize outcomes while minimizing treatment-related morbidity in medically complex patients. 

## Conclusions

This case underscores that DLBCL can present with concurrent pulmonary and cervical involvement, closely mimicking primary lung malignancy or metastatic disease, where overlapping conditions can mask serious disease. It highlights the importance of maintaining diagnostic vigilance when the clinical picture remains incongruent, as seen in this patient presenting in the setting of advanced liver disease and multiple comorbidities. When clinical and radiologic suspicion for malignancy persists despite an initial nondiagnostic biopsy, repeat or alternative tissue sampling should be pursued without delay, as a single negative biopsy does not reliably exclude aggressive lymphoma. Awareness of these rare presentations is essential, as early recognition and accurate tissue diagnosis are critical for the timely initiation of appropriate therapy in this aggressive yet potentially curable lymphoma.

## References

[1] Armitage JO, Gascoyne RD, Lunning MA, Cavalli F (2017). Non-Hodgkin lymphoma. Lancet.

[2] Xie Y, Pittaluga S, Jaffe ES (2015). The histological classification of diffuse large B-cell lymphomas. Semin Hematol.

[3] Padala SA, Kallam A (2023). Diffuse large B-cell lymphoma. StatPearls.

[4] Xu H, Xu K, Wang R, Liu X (2015). Primary pulmonary diffuse large B-cell lymphoma on FDG PET/CT-MRI and DWI. Medicine (Baltimore).

[5] Karki SS, Pandey S, Karki S, Paudel D, Pathak C (2025). Diffuse large B-cell lymphoma of the thyroid in a patient with Hashimoto thyroiditis: a diagnostic dilemma. Clin Case Rep.

[6] Kesireddy M, Lasrado S (2024). Thyroid lymphoma. StatPearls.

[7] Li S, Young KH, Medeiros LJ (2018). Diffuse large B-cell lymphoma. Pathology.

[8] Castillo JJ, Winer ES, Olszewski AJ (2014). Sites of extranodal involvement are prognostic in patients with diffuse large B-cell lymphoma in the rituximab era: an analysis of the Surveillance, Epidemiology and End Results database. Am J Hematol.

[9] Stein SA, Wartofsky L (2013). Primary thyroid lymphoma: a clinical review. J Clin Endocrinol Metab.

[10] Sanguedolce F, Zanelli M, Zizzo M (2021). Primary pulmonary B-cell lymphoma: a review and update. Cancers (Basel).

[11] Peng Y, Qi W, Luo Z (2022). Role of 18F-FDG PET/CT in patients affected by pulmonary primary lymphoma. Front Oncol.

[12] Tongbai T, McDermott S, Kiranantawat N, Muse VV, Wu CC, Shepard JA, Gilman MD (2019). Non-diagnostic CT-guided percutaneous needle biopsy of the lung: predictive factors and final diagnoses. Korean J Radiol.

[13] Tang J, Yang B, Bai Y (2025). Diagnostic challenges and management strategies of pulmonary mucosa-associated lymphoid tissue lymphoma: a case report and literature review. Front Med (Lausanne).

[14] Fujioka N, Kai Y, Kataoka R (2023). Primary pulmonary diffuse large B-cell lymphoma presenting multiple nodules mimicking metastasis: a case report. Respirol Case Rep.

[15] Li S, Huang Y, Wang L, Zhou J (2025). Primary pulmonary diffuse large B cell lymphoma presenting with features of organizing pneumonia: a case report. J Med Case Rep.

[16] Sharma A, Jasim S, Reading CC (2016). Clinical presentation and diagnostic challenges of thyroid lymphoma: a cohort study. Thyroid.

[17] Walsh S, Lowery AJ, Evoy D, McDermott EW, Prichard RS (2013). Thyroid lymphoma: recent advances in diagnosis and optimal management strategies. Oncologist.

[18] Silkenstedt E, Salles G, Campo E, Dreyling M (2024). B-cell non-Hodgkin lymphomas. Lancet.

[19] Krens SD, Lassche G, Jansman FGA (2019). Dose recommendations for anticancer drugs in patients with renal or hepatic impairment. Lancet Oncol.

